# Supplementary material for: Ensemble Models for Tick Vectors: Standard Surveys Compared with Convenience Samples
Source: Diseases. 2022 Jun 8;10(2):32. doi: 10.3390/diseases10020032 (PMC9222110; doi:10.3390/diseases10020032)

Supplemental Table S1. Performance metrics associated with the five modeling algorithms for each species. Threshold is the value of the continuous score used to delineate presence from absence when sensitivity = specificity. AUC is the area under the Receiver Operator Characteristic curves. Accuracy is the ratio of sum of correctly predicted presence and absence to the sample size from cross validations.

Amblyomma americanum	Threshold	AUC	SE	Accuracy CV
Logistic	0.26	0.80	0.05	0.96
BRT	0.28	0.72	0.04	0.92
RF	0.24	0.72	0.05	0.93
MARS	0.27	0.75	0.04	0.83
MaxEnt	0.35	0.77	0.04	0.96

Ixodes scapularis	Threshold	AUC	SE	Accuracy CV
Logistic	0.22	0.78	0.03	0.82
BRT	0.29	0.70	0.04	0.88
RF	0.25	0.74	0.04	0.90
MARS	0.27	0.65	0.04	0.83
MaxEnt	0.34	0.79	0.04	0.84

Supplemental Table S2. Omission error sites of convenience samples with location, URL to imagery, distance to nearest standard SDM location and details of location.

			Ixodes scapularis convenience samples		
**ID**	**Lat.**	**Long.**	**Image**	**Dist. SDM (m)**	**Local Site**
23	30.284110	-84.151350	https://www.google.com/maps/place/30%C2%B017'02.8%22N+84%C2%B009'04.9%22W/@30.2840492,-84.1514955,97m/data=!3m1!1e3!4m5!3m4!1s0x0:0x0!8m2!3d30.28411!4d-84.15135	100	parking lot
31	30.211416	-84.749755	https://www.google.com/maps/place/30%C2%B012'41.1%22N+84%C2%B044'59.1%22W/@30.211416,-84.7519437,777m/data=!3m2!1e3!4b1!4m5!3m4!1s0x0:0x0!8m2!3d30.211416!4d-84.749755	100	OK?
121	30.082560	-84.171700	https://www.google.com/maps/place/30%C2%B004'57.2%22N+84%C2%B010'18.1%22W/@30.08256,-84.1760774,1557m/data=!3m1!1e3!4m5!3m4!1s0x0:0x0!8m2!3d30.08256!4d-84.1717	100	OK? Mudflat at low tide
128	30.053600	-84.530000	https://www.google.com/maps/place/30%C2%B003'13.0%22N+84%C2%B031'48.0%22W/@30.0545565,-84.528337,779m/data=!3m1!1e3!4m5!3m4!1s0x0:0x0!8m2!3d30.0536!4d-84.53	100	OK? How far SW Sopchoppy?
240	28.037400	-81.799800	https://www.google.com/maps/place/28%C2%B002'14.6%22N+81%C2%B047'59.3%22W/@28.0374,-81.8085547,3176m/data=!3m1!1e3!4m5!3m4!1s0x0:0x0!8m2!3d28.0374!4d-81.7998	100	poorly defined region
284	25.192680	-80.355360	https://www.google.com/maps/place/25%C2%B011'33.7%22N+80%C2%B021'19.3%22W/@25.19268,-80.4253978,26047m/data=!3m1!1e3!4m5!3m4!1s0x0:0x0!8m2!3d25.19268!4d-80.35536	100	OK? On roadside
DPI 11	27.886344	-82.389488	Old US 41 & Burts Rd (SE corner of), Tampa - Bing	100	OK? Intersect but gives corner

DP 12	28.282292	-80.607286	South 24th Street; Cocoa Beach - Bing	100	OK? Single house; short street segment
129	30.047900	-84.417100	https://www.google.com/maps/place/30%C2%B002'52.4%22N+84%C2%B025'01.6%22W/@30.0443894,-84.4210268,3115m/data=!3m1!1e3!4m5!3m4!1s0x0:0x0!8m2!3d30.0479!4d-84.4171	141	OK?
134	30.034730	-84.462150	https://www.google.com/maps/place/30%C2%B002'05.0%22N+84%C2%B027'43.7%22W/@30.0366085,-84.4653982,1557m/data=!3m1!1e3!4m5!3m4!1s0x0:0x0!8m2!3d30.03473!4d-84.46215	141	OK?
237	28.043400	-82.266500	https://www.google.com/maps/place/28%C2%B002'36.2%22N+82%C2%B015'59.4%22W/@28.0434,-82.2752547,3176m/data=!3m1!1e3!4m5!3m4!1s0x0:0x0!8m2!3d28.0434!4d-82.2665	141	OK?
DPI 1	28.019187	-82.133439	507 Whitehall St, Tampa - Bing	141	OK? only address on street; wooded habitat on property
37	30.194527	-84.766672	https://www.google.com/maps/place/30%C2%B011'40.3%22N+84%C2%B046'00.0%22W/@30.1926631,-84.767584,728m/data=!3m1!1e3!4m5!3m4!1s0x0:0x0!8m2!3d30.194527!4d-84.766672	200	center line
38	30.196688	-84.727574	https://www.google.com/maps/place/30%C2%B011'48.1%22N+84%C2%B043'39.3%22W/@30.196688,-84.7297627,777m/data=!3m2!1e3!4b1!4m5!3m4!1s0x0:0x0!8m2!3d30.196688!4d-84.727574	200	OK? Intersect

40	30.181999	-84.754825	https://www.google.com/maps/place/30%C2%B010'55.2%22N+84%C2%B045'17.4%22W/@30.1821068,-84.754766,97m/data=!3m1!1e3!4m13!1m7!3m6!1s0x0:0x0!2zMzDCsDEwJzU1LjIiTiA4NMKwNDUnMTcuNCJX!3b1!8m2!3d30.181999!4d-84.754825!3m4!1s0x0:0x0!8m2!3d30.181999!4d-84.754825	200	OK?
260	27.806805	-81.498938	https://www.google.com/maps/place/27%C2%B048'24.5%22N+81%C2%B029'56.2%22W/@27.806805,-81.6390137,50921m/data=!3m1!1e3!4m5!3m4!1s0x0:0x0!8m2!3d27.806805!4d-81.498938	200	multiple lots; area to West of selected site
27	30.239313	-84.269727	https://www.google.com/maps/place/30%C2%B014'21.5%22N+84%C2%B016'11.0%22W/@30.2385715,-84.2895539,6217m/data=!3m1!1e3!4m5!3m4!1s0x0:0x0!8m2!3d30.239313!4d-84.269727	224	road center
153	30.030100	-84.408300	https://www.google.com/maps/place/30%C2%B001'48.4%22N+84%C2%B024'29.9%22W/@30.0291154,-84.4093622,389m/data=!3m1!1e3!4m5!3m4!1s0x0:0x0!8m2!3d30.0301!4d-84.4083	224	poorly defined region centroid of parcel
263	27.468300	-82.695500	https://www.google.com/maps/place/27%C2%B028'05.9%22N+82%C2%B041'43.8%22W/@27.4683,-82.6976887,798m/data=!3m2!1e3!4b1!4m5!3m4!1s0x0:0x0!8m2!3d27.4683!4d-82.6955	224	geocoded to Tampa Bay

281	25.221430	-81.082350	https://www.google.com/maps/place/25%C2%B013'17.2%22N+81%C2%B004'56.5%22W/@25.22143,-81.1523878,26040m/data=!3m1!1e3!4m5!3m4!1s0x0:0x0!8m2!3d25.22143!4d-81.08235	224	location insufficient but forests occur there
DPI 2	27.201662	-81.930490	Bing Maps - Directions, trip planning, traffic cameras & more	224	intersect
22	30.294700	-84.185100	https://www.google.com/maps/place/30%C2%B017'40.9%22N+84%C2%B011'06.4%22W/@30.2947,-84.2201189,12427m/data=!3m1!1e3!4m5!3m4!1s0x0:0x0!8m2!3d30.2947!4d-84.1851	283	uncertain location
269	26.003000	-81.605200	https://www.google.com/maps/place/26%C2%B000'10.8%22N+81%C2%B036'18.7%22W/@26.003,-81.6227095,6468m/data=!3m1!1e3!4m5!3m4!1s0x0:0x0!8m2!3d26.003!4d-81.6052	283	vague location; midline of road
1	30.811720	-85.226460	https://www.google.com/maps/place/30%C2%B048'42.2%22N+85%C2%B013'35.3%22W/@30.81172,-85.2439695,5787m/data=!3m1!1e3!4m5!3m4!1s0x0:0x0!8m2!3d30.81172!4d-85.22646	316	Visitors Center
200	29.611081	-82.921459	https://www.google.com/maps/place/29%C2%B036'39.9%22N+82%C2%B055'17.3%22W/@29.5956332,-82.9290121,6257m/data=!3m1!1e3!4m5!3m4!1s0x0:0x0!8m2!3d29.611081!4d-82.921459	316	OK?

4	30.772870	-85.216360	https://www.google.com/maps/place/30%C2%B046'22.3%22N+85%C2%B012'58.9%22W/@30.77287,-85.2338695,5789m/data=!3m1!1e3!4m5!3m4!1s0x0:0x0!8m2!3d30.77287!4d-85.21636	361	in the water
124	30.069200	-84.388900	https://www.google.com/maps/place/30%C2%B004'09.1%22N+84%C2%B023'20.0%22W/@30.0619574,-84.3965389,3114m/data=!3m1!1e3!4m5!3m4!1s0x0:0x0!8m2!3d30.0692!4d-84.3889	412	How far south of Carraway Cutoff; which side of road?
274	25.444000	-80.466300	https://www.google.com/maps/place/25%C2%B026'38.4%22N+80%C2%B027'58.7%22W/@25.4514403,-80.5090437,12996m/data=!3m1!1e3!4m5!3m4!1s0x0:0x0!8m2!3d25.444!4d-80.4663	412	intersect major roads
DPI 3	27.733053	-80.441699	Hobart Park, NW Cor. of 58 Ave. & 77 Street, Wabasso, fl - Google Maps	424	Intersect and codes to park building
DPI 4	27.018985	-82.275025	North Port - Bing Maps	447	road segment 800 m; vague address; typo - North Port, FL
143	30.032570	-84.490390	https://www.google.com/maps/place/30%C2%B001'57.3%22N+84%C2%B029'25.4%22W/@30.0346413,-84.4946923,779m/data=!3m1!1e3!4m5!3m4!1s0x0:0x0!8m2!3d30.03257!4d-84.49039	447	multiple sand tracks

199	29.605349	-83.124611	https://www.google.com/maps/place/29%C2%B036'19.3%22N+83%C2%B007'28.6%22W/@29.6060579,-83.1255337,782m/data=!3m1!1e3!4m5!3m4!1s0x0:0x0!8m2!3d29.605349!4d-83.124611	447	which woodlot?; poorly defined region centroid of parcel
277	25.394510	-80.617680	https://www.google.com/maps/place/25%C2%B023'40.2%22N+80%C2%B037'03.7%22W/@25.3957506,-80.7028241,52006m/data=!3m1!1e3!4m5!3m4!1s0x0:0x0!8m2!3d25.39451!4d-80.61768	447	hammock multiple; N or S side of Rte 9336?
3	30.806390	-85.205610	https://www.google.com/maps/place/30%C2%B048'23.0%22N+85%C2%B012'20.2%22W/@30.80639,-85.2099874,1447m/data=!3m1!1e3!4m5!3m4!1s0x0:0x0!8m2!3d30.80639!4d-85.20561	500	uncertain location
46	30.151120	-84.241370	https://www.google.com/maps/place/30%C2%B009'04.0%22N+84%C2%B014'28.9%22W/@30.15112,-84.2588795,6223m/data=!3m1!1e3!4m5!3m4!1s0x0:0x0!8m2!3d30.15112!4d-84.24137	500	1.5 km from intersect; geocode centroid of property
241	28.012800	-82.263900	https://www.google.com/maps/place/28%C2%B000'46.1%22N+82%C2%B015'50.0%22W/@28.0128,-82.2682774,1588m/data=!3m1!1e3!4m5!3m4!1s0x0:0x0!8m2!3d28.0128!4d-82.2639	500	poorly defined region
268	26.976300	-81.945600	https://www.google.com/maps/place/26%C2%B058'34.7%22N+81%C2%B056'44.2%22W/@26.9763,-81.9806189,12826m/data=!3m1!1e3!4m5!3m4!1s0x0:0x0!8m2!3d26.9763!4d-81.9456	500	marsh

DPI	30.481353	-86.199822	692 Piney Point Rd, Freeport, fl - Bing Maps	510	business address; large plot of forested land,
150	30.020300	-84.408700	https://www.google.com/maps/place/30%C2%B001'13.1%22N+84%C2%B024'31.3%22W/@30.0195986,-84.4170417,3115m/data=!3m1!1e3!4m5!3m4!1s0x0:0x0!8m2!3d30.0203!4d-84.4087	566	poorly defined region centroid of parcel
265	27.199700	-81.988100	https://www.google.com/maps/place/27%C2%B011'58.9%22N+81%C2%B059'17.2%22W/@27.1997,-82.1281757,51203m/data=!3m1!1e3!4m5!3m4!1s0x0:0x0!8m2!3d27.1997!4d-81.9881	608	on bridge over river
DPI 5	27.346740	-80.332534	810 Kitterman Rd, Fort Pierce, fl - Bing	608	geocode to only house on plot
DPI 6	27.360310	-80.489204	Peacock Rd, Fort Pierce. fl - Bing	608	road center farmland
DPI 7	25.588039	-80.459183	16300 SW 184 St, Miami, fl - Bing	806	House on SW 192 but address is 184th
266	27.166000	-80.687000	https://www.google.com/maps/place/27%C2%B009'57.6%22N+80%C2%B041'13.2%22W/@27.166,-80.8270757,51219m/data=!3m1!1e3!4m5!3m4!1s0x0:0x0!8m2!3d27.166!4d-80.687	860	in the field but locale uncertain

39	30.174945	-85.623249	https://www.google.com/maps/place/30%C2%B010'29.8%22N+85%C2%B037'23.7%22W/@30.174945,-85.6407585,5825m/data=!3m1!1e3!4m5!3m4!1s0x0:0x0!8m2!3d30.174945!4d-85.623249	894	center line W bound E15th St
DPI 8	26.989992	-82.130409	766+Merrick+Ln+NW,+PORT+CHARLOTTE,+FL+33948 - Bing Maps	1000	OK?
238	28.038100	-82.173300	https://www.google.com/maps/place/28%C2%B002'17.2%22N+82%C2%B010'23.9%22W/@28.0381,-82.1908095,6352m/data=!3m1!1e3!4m5!3m4!1s0x0:0x0!8m2!3d28.0381!4d-82.1733	1105	pasture
DPI 9	27.267876	-82.326286	5703 Howard Creek Rd, Sarasota, fl - Bing	1140	farm house; forest to NE
DPI 10	27.886591	-82.059677	8549 Carey Rd, Keysville, fl - Bing	1640	farm house; near Alafia River

			Amblyomma americanum convenience samples		
**ID**	**Lat**	**Long.**	**Image**	**Dist. SDM (m)**	**Local Site**
**DPI1A**	28.422339	-80.585442	Maca Rd (at gate N of S Patrol Rd) cape canaveral - Bing	141	Street intersection
**DPI2A**	26.726818	-80.059035	1508 Division Ave west palm beach, fl - Bing Maps	224	OK?
**DPI3A**	28.264856	-81.424484	1448 Flamingo Blvd Kissimmee, fl - Bing	283	OK?
**DPI4A**	27.214910	-80.794641	3202 SE 33rd Terrace, okechobee, fl - Bing	700	Distance to Okeechobee Battlefield State Park; coll 1989
**DPI5A**	27.293056	-80.254572	9416 Gumbo Limbo Ln, jensen beach - Bing	1200	Inholding to state park
**DPI6A**	28.751011	-82.535471	35 Linder Circle homosassa, fl - Bing	1432	1.1 miles Homosassa WMG area
**DPI7A**	27.910614	-81.568062	916 Primrose Way, lake wales, fl - Bing	2927	Pine plantation in back yard
**DPI8A**	26.982799	-80.111567	18231 SE Island Dr tequesta, fl - Bing	14009	South end JD State Park

Supplemental Figure S1. Sites of tick surveys in Florida. *A. americanum* (**A**,**B**). (**A**) shows standard survey locations of *A*. *americanum* collected (filled circle) or never collected (open circle). (**B**) shows convenience locations from lizards (filled circle) or from public surveys (open circle). *I. scapularis* (**C**,**D**). (**C**) shows standard survey locations of *I. scapularis* collected (filled circle) or never collected (open circle). (**D**) shows convenience locations from lizards (open circle) or from public surveys (filled circle).

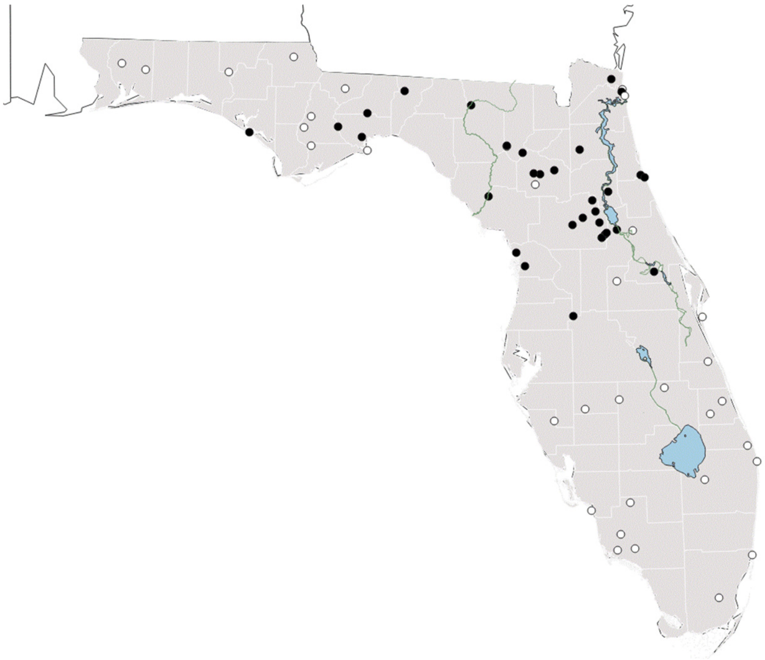

B

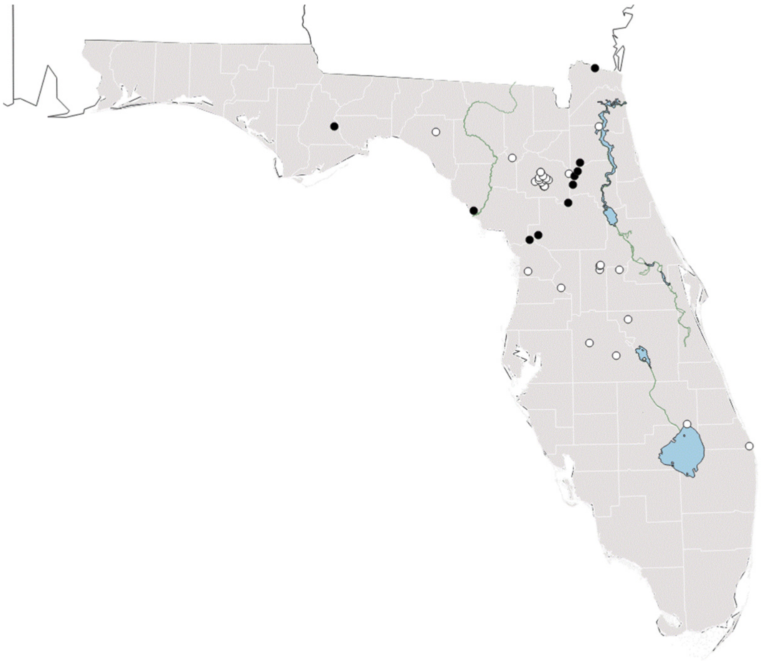

C

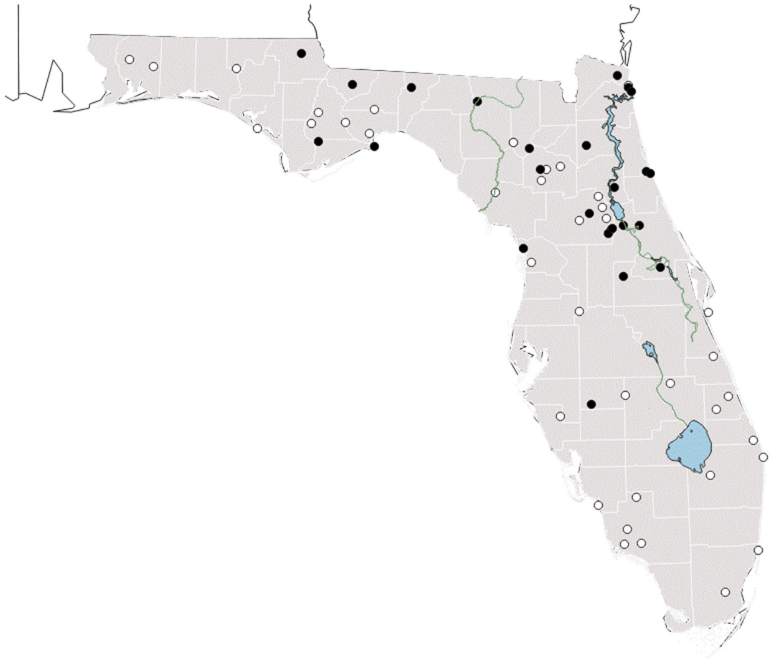

D

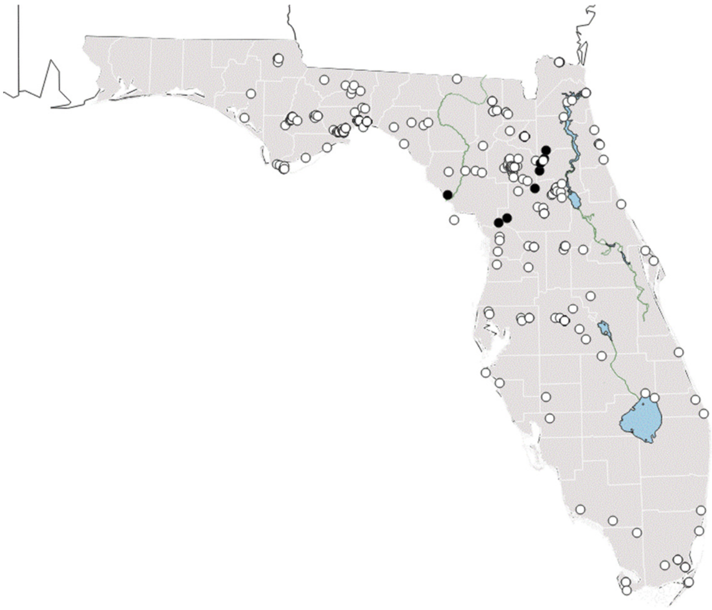

Supplement: Supplementary file 1 [file diseases-10-00032-s001.zip › diseases-1650698-supplementary.pdf]
